# *Sporades jaechi* sp. nov. with comments on classification of the New Caledonian genus *Sporades* Fauvel (Coleoptera, Carabidae, Trechini, Trechodina)

**DOI:** 10.3897/zookeys.908.48707

**Published:** 2020-02-03

**Authors:** James K. Liebherr

**Affiliations:** 1 Department of Entomology, John H. and Anna B. Comstock Hall, 129 Garden Ave., Cornell University, Ithaca, NY 14853-2601, USA Cornell University Ithaca United States of America

**Keywords:** aedeagus, genitalia, phylogenetics, taxonomy

## Abstract

*Sporades jaechi***sp. nov.** from Poum, New Caledonia is newly described and shown to be a member of the monophyletic Sporades subgenus Perileptosporades Deuve, 2010. *Sporades millei* Giachino and *S. schuhi* Donabauer are newly recognized as members of the subgenus Perileptosporades, and a key to its species is provided. Although *Perileptosporades* can be defined monophyletically relative to the rest of the genus, genitalic variation among species assigned to the nominate subgenus Sporades Fauvel, 1882 leave monophyly of that taxon ambiguously supported. Several morphological characters of long- standing use have been proposed to define the mutual monophyly of *Sporadess. l.* and its putative adelphotaxon, *Trechodes* Blackburn, 1901. Increasing knowledge concerning the diversity of male genitalic characters among *Sporades* spp. lends support to a recently proposed molecular phylogenetic hypothesis positing that *Sporades* evolved from within *Trechodes*. The consequences of the alternate phylogenetic hypotheses on their attendant nomenclature are discussed. An additional locality record for *S. sexpunctatus* Fauvel expands the known distribution of this species to include most of Grande Terre, New Caledonia.

## Introduction

Fauvel established *Sporades* for his species *S. sexpunctatus* (Fauvel 1882), placing the new genus in the tribe Trechini while noting that the elytra are basally grooved as in Pogonini, the apical palpomeres are narrowly cylindrical similar to those of Bembidiini, and the frontal grooves are very deep as in *Trechus* Clairville, 1806. Jeannel (1926) established a taxonomic framework for the World's trechine lineages, proposing Trechodini at the tribal level within a subfamily Trechinae, with *Sporades* placed next to *Trechodes* Blackburn, 1901. Trechodines were defined by Jeannel based on tridentate mandibles (mola, premolar tooth, and retinaculum present) with these projections obtuse; anterior tibia without an external apical spine; and male aedeagal median lobe with the dorsal surface membranous, the base not enclosed into a bulb.

*Sporades* remained monotypic until Uéno (1966) described two additional species. He noted the great similarity of the three species to *Trechodes*, suggesting that *Sporades* “is so close to Trechodes that it may be regarded as a subgenus of the latter (Uéno 1966: 29). ” Over the past 10 years, three more contributions have added eight names to the described fauna: Deuve (2010) described three new species; Donabauer (2011), two new species; and Giachino (2012), three new species. Deuve (2010) proposed *Perileptosporades* as a new subgenus, *S. theryi* Deuve as the type species, defining its monophyly by the labrum trilobed, i.e., the median excavation bearing a small tooth or broader protuberance at its deepest point; pronotum narrow and the median base straight, not extended posteriorly between the hind angles; and the pronotal lateral setae placed far forward near the pronotal front angles. The latter two authors were not aware of Deuve’s (2010) publication, and so did not attempt species placement relative to subgenus Perileptosporades.

This contribution adds one more new species to the New Caledonian *Sporades* fauna. The new species described below is placed in subgenus Perileptosporades, with a preliminary assessment of relationships of the five species assignable to that subgenus provided. Cladistic relationships of the two *Sporades* subgenera, and *Sporadess. l.* relative to *Trechodes* are discussed in light of information derived from morphological characters and molecular sequence data (Maddison et al. 2019), illustrating that complex interactions may result from linking taxonomic nomenclature to hypotheses of phylogenetic history. An additional locality for *S. sexpunctatus* Fauvel is reported, expanding the known geographical distribution of this species.

## Materials and methods

### Taxonomic material

This contribution is based on two specimens received on loan from the Naturhistorisches Museum Wien, Austria (**NHMW**). Based on permit requirements in force at the time of collection, the holotype of the *Sporades jaechi* sp. nov. is deposited in the Vienna Museum.

### Laboratory techniques

Dissection techniques and macro-photographic procedures follow those of Liebherr (2015). The very small aedeagus of *Sporades* spp. requires a line drawing to best represent that structure, and so the illustration presented below follows the orientation used in previous treatments (Uéno 1966, Deuve 2010, Donabauer 2011, Giachino 2012).

Body length was measured as the distance from the mandibular apices to the elytral apex, measured in linear segments along the dorsal body midline. Body measurement abbreviations follow Uéno (1966):

**EL** elytral length as above;

**EW** maximal elytral width;

**HW** head width;

**PA** apical width of pronotum measured between anteriormost margins of front angles; PB, basal width of pronotum;

**PW** maximal pronotal width;

**PL** median length of pronotum.

Terminology for trechine anatomy follows Jeannel (1926) and for male genitalia, Deuve (1993).

## Taxonomy

### Sporades (Perileptosporades) jaechi sp. nov.

Taxon classificationAnimaliaColeopteraCarabidae

D7E089CE-24C7-5EAE-9507-BFA9F8F2E1DF

http://zoobank.org/FADB464E-1B3D-465A-B99E-5DCD68EC96CA

Figures 1 , 2–4 , 5

#### Type material.

Holotype male, point mounted, dissected, apical three abdominal ventrites glued onto point (NHMW), labelled: NEW CALEDONIA (NC 27) / N-Prov., Poum Com. / unnamed river / 9.XI.2016, leg. M.A. Jäch // ca. 7 km NE Poum / ca. 15 m a.s.l. / 20°12'18.3"S 164°5'27.7"/ almost dried out lowland river // HOLOTYPE / Sporades / jaechi / J.K. Liebherr 2019 (black-margined red label) // polyethylene genitalia vial on pin.

#### Diagnosis.

**Figure 1.**
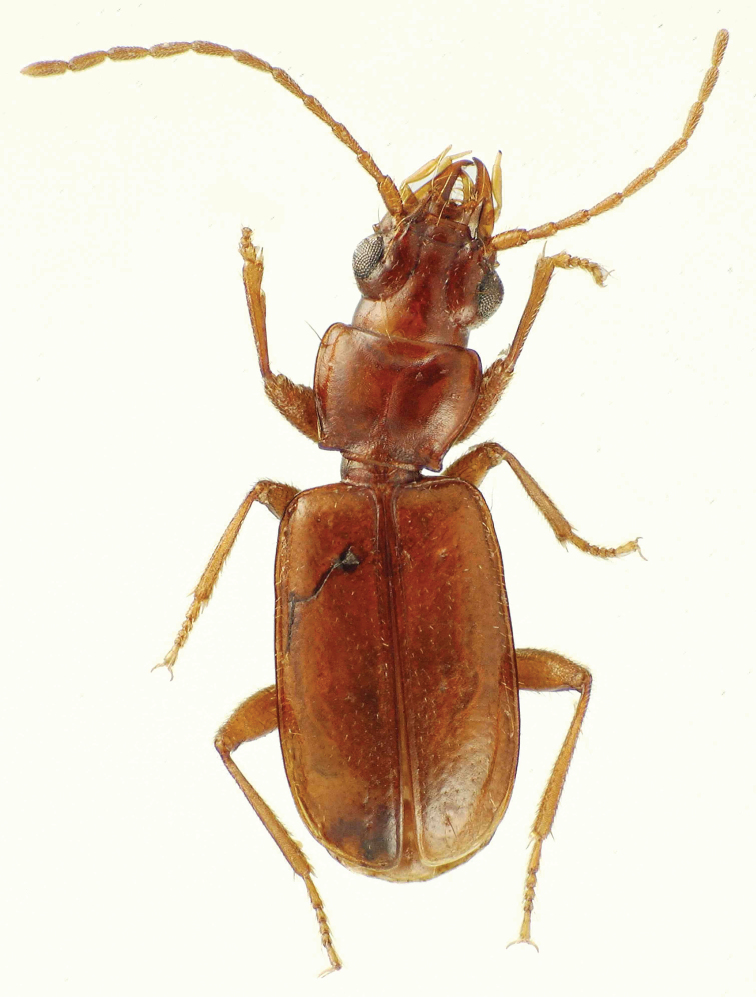
*Sporades jaechi* sp. nov. holotype male; body length 3.0 mm.

Assignable to the subtribe Trechodina based on the tridentate mandibles bearing an obtuse tooth between the mola and retinaculum (Jeannel 1926: 396), and the male aedeagus with a membranous dorsal surface, the basal sclerotization limited to lateral apophyses (Fig. 4). This species is placeable in *Sporades* based on the little-convex, sparsely setose elytral surface, the quadrate pronotum with basal margin little protruded and unbordered medially, and the basal male protarsomere alone dilated (Uéno 1966). This species is assignable to subgenus Perileptosporades based on the trilobed labrum, with a well-developed median tooth in the apical excavation, and the lateral pronotal setae placed very near the front angles (Fig. 1; Deuve 2010: figs 2, 3; Donabauer 2011: fig. 1). Among the five sg. Perileptosporades spp., this species shares the glabrous pronotum with S. (P.) modestior Deuve and S. (P.) schuhi Donabauer. However, this species can be diagnosed from both by the convex pronotal anterior marginal collar associated with the well-developed, arcuate anterior transverse impression (Fig. 1). In contrast, S. (P.) schuhi has the anterior transverse impression obsolete with the medioanterior portion of the pronotum flat, and S. (P.) modestior has the anterior transverse impression shallower, meeting the median impression at an angulate juncture.

#### Description.

Male holotype body length 3.0 mm from mandibular apex to elytral apex. Head, pronotum, elytra and femora ferruginous, maxillary and labial palpi flavous, all antennomeres rufoflavous, and tibiae and tarsomeres rufobrunneous; head and pronotum with well-developed microsculpture, isodiametric to transversely stretched isodiametric medially on frons, vertex and pronotal disc, more transverse on ocular lobes and lateral margins of pronotum; elytra glossy, transverse sculpticells partially covering convexities surrounding depressions associated with setal insertions of elytral pelage.

Head broad, HW/PW = 0.92, eyes convex but outer curvature consistent with that of ocular lobe behind eye; frontal grooves deepest between hind margins of eyes and anterad near fronto-clypeal suture; two supraorbital setae; four clypeal setae; labrum deeply and arcuately excavate medially, with a distinct obtuse tooth along midline of excavation; mandibles elongate, distance from dorsal condyle of left mandible to mandibular apex ~ 2 × distance from condyle to anterolateral apex of labrum; antennae filiform, antennomere IX length 4× maximal breadth; submentum with six setae arcuately arranged across width of convexity fused to gula; mentum with oblique longitudinal depressions terminated posterad mentum setae, and with acute median tooth bearing a longitudinal median groove; ligula convex medially, paraglossae porrect, elongate (Jeannel 1926: fig. 285); maxillary palpomeres elongate, penultimate palpomere expanded on medial surface, length 2.2 × length of narrow, spindle-like apical palpomere; all antennomeres setose; vertex with sparse setal pelage, the setal insertions not disturbing surface, genae more densely setose posterad eyes, and eyes sparsely setose, the setae shorter; ventral surface of head capsule glabrous except for one macroseta at ventral terminus of groove defining ocular lobe, and sparse short setae anterad groove near maxillary fossa.

Pronotum moderately broad, PW/PL = 1.31; base constricted, PA/PB = 1.11, PW/PB = 1.32, hind angles narrowly rounded, protuberant, basal pronotal setae set on concave margin anterad angle; median base straight, forming a transverse collar extended posterad finely margined lateral lobes bearing hind angles, the base finely margined across width, isolated from disc by broad, well-developed basal transverse impression that is deepest and narrowest anterad carinate hind pronotal margin mesad hind angles; median longitudinal impression fine and shallow on disc; anterior transverse impression broad and deep, extended to front margin mesad front angles and defining a broadly convex anterior collar that is distinguished from the disc by its glossy surface due to very shallowly margined sculpticells; front angles nearly imperceptibly protruded, the pronotal margin rounded there to the narrow lateral marginal depression; anterior pronotal setae placed far forward along margin, 0.19 × distance from front posterad to hind angle.

Elytra elongate, quadrate, lateral margins nearly parallel at midlength, the elytra broadest near apical 1/4, EW/EL = 1.62; disc flat to slightly depressed laterad elevated sutural interval; parascutellar seta present mesad base of stria 1, equidistant from stria and basal groove; basal groove slightly recurved from sutural stria to broadly rounded humeri; lateral marginal depression narrow but with sculpticells visible at depth, depression broadest anterad very short recurrent stria, elytral margin slightly concave there; striae 2 and 3 traceable on disc, though depressions are obscured by punctures associated with elytral setal pelage, outer striae not visible; two dorsal elytral setae present in third stria, plus a third posterior seta present mesad apex of very shallow, difficult to trace recurrent stria; subapical and apical setae present, former at base of recurrent stria, latter inside marginal bead near rounded elytral apex; lateral elytral setae arranged as four anterior setae posterad humerus, two setae isolated ~ 0.650.70 × elytral length, and two setae laterad recurrent stria at ~ 0.90 × elytral length; elytral surface covered with setal pelage, the setae as long as the distances between them, their arrangement tending to longitudinal series of setae, especially traceable in the sutural stria; metathoracic flight wings fully developed with complete venation.

Ventral surface bearing both macrosetae and a shorter setal pelage; prosternum with setal pelage medially and a transverse series of ten macrosetae along anterior margin; metathoracic ventrites and metacoxae with setal pelage; abdominal ventrites with setal pelage of similar development to that of elytra; apical ventrite of male with one seta on each side along apical margin which is slightly concave along midline.

#### Male genitalia.

**Figures 2–4.**
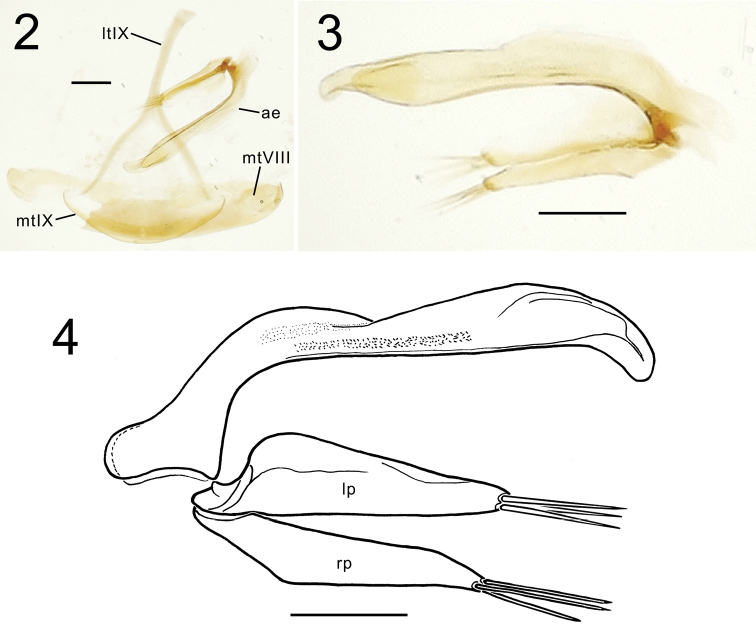
Male aedeagus of *Sporades jaechi* holotype: **2** aedeagus *in situ* with laterotergite IX and tergites of abdominal segments VIII and IX **3** male aedeagus, dextral view **4** male aedeagus, sinistral view. Abbreviations: ae, aedeagus; lt IX, laterotergite IX; mt VIII, mediotergite VIII; mt IX, mediotergite IX; lp, left paramere; rp; right paramere. Scale bars 0.10 mm.

(*N* = 1). Aedeagus lightly sclerotized, small relative to large, λ-shaped antecostal apodeme of abdominal segment IX (Fig. 2); median lobe elongate with broad, broadly downturned apex (Figs 3, 4), apex bearing furrow on left side associated with ostial opening (Fig. 4); internal sac without evidence of sclerites, although spicules on sac surface visible near midlength in non-everted dissection; parameres robustly defined, left broader basally with rounded apex, right narrower overall, both with three apical setae.

#### Etymology.

This species is named to honor the collector of the unique holotype, Dr. Manfred Jäch; a noted world authority on water beetle systematics and biodiversity.

#### Distribution and habitat.

**Figure 5.**
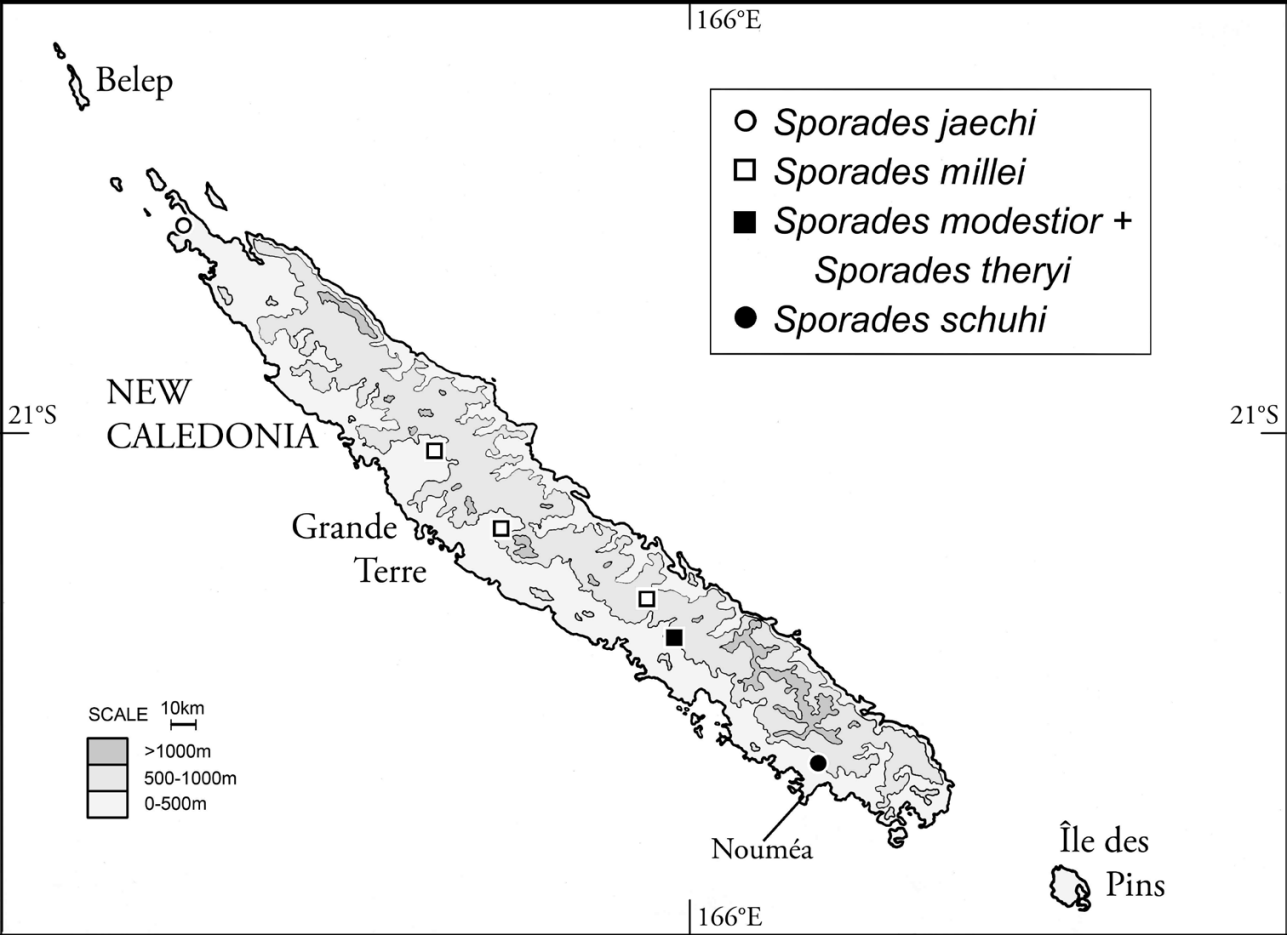
Distributional records for species of subgenus Sporades species of subgenus Perileptosporades.

Known only from northernmost Grande Terre (Fig. 5). The almost dried out river at the type locality is approximately “5–7 m wide, in dense forest, with residual pools, substrate: sand; most specimens collected from submerged leaf packs (covered by sand and mud) at margin of residual pool (~ 5 × 10 m) (M. Jäch, pers. comm.)”.

### Key to the adults of Sporades sg. Perileptosporades Deuve

This key is based on information presented in Deuve (2010), Donabauer (2011) and Giachino (2012) complemented by the specimen of the new species described herein. Even though the types of the prior names have not been viewed, the comprehensive descriptions of prior authors allow presentation of a provisional key to assist identification of additional specimens. *Sporades schuhi* Donabauer and *S. millei* Giachino are newly assigned to sg. Perileptosporades below, as the authors were not aware of the description of that subgenus by Deuve (2010).

**Table:** 

1	Pronotum glabrous or very sparsely setose, lateral marginal depression narrow	**2**
–	Pronotum densely setose, covered with a pelage of short setae, lateral marginal depression broad	***Sporades theryi* Deuve**
2	Pronotum either with a shallow anterior transverse impression, or the impression obsolete, the medioanterior portion of pronotal disc not upraised in a distinct collar; male aedeagal apex narrow dorsoventrally, the dorsal and ventral margins subparallel basad the narrowly rounded tip (Deuve 2010: fig. 5; Donabauer 2011: fig. 1B)	**3**
–	Pronotal anterior transverse impression deep, broad, arcuate, defining a glossy, raised anterior collar (Fig. 1); male aedeagal apex broader dorsoventrally, tip broadly rounded (Fig. 4)	***Sporades jaechi* sp. nov.**
3	Elytral recurrent vein very reduced to obsolete, apical elytral margin not interrupted; aedeagal median lobe distinctly downcurved, apex extended beyond straight shaft of lobe ~ 2× dorsoventral breadth (Deuve 2010: fig. 5; Giachino 2012: figs 8, 9)	**4**
–	Elytra with evident, short recurrent vein that interrupts apical elytral margin; aedeagal median lobe slightly downcurved, apex extended beyond straight shaft of lobe ~ 3× dorsoventral breadth (Donabauer 2011: fig. 1B)	***Sporades schuhi* Donabauer**
4	Pronotum glabrous except for lateral and basal macrosetae (Deuve 2010: fig. 3 [*sic* fig. 2, p. 63); apex of male median lobe distinctly downturned, the apical portion extended 2 × dorsoventral breadth, shaft broader dorsoventrally just apicad midlength	***Sporades modestior* Deuve**
–	Pronotum with sparse pelage of very short setae accompanying lateral and basal macrosetae (Giachino 2012: fig. 2); apex of male aedeagal median lobe only slightly downturned to somewhat more downturned, shaft uniformly narrow along length	***Sporades millei* Giachino**

### Sporades (s. s.) sexpunctatus

Taxon classificationAnimaliaColeopteraCarabidae

Fauvel, 1882

5C532D98-85BE-5C96-8AED-B55429559273

Figure 6

#### Diagnosis.

This species is easily diagnosed among all *Sporades* by the male aedeagus, wherein the median lobe is expanded and twisted apically (Jeannel 1926: fig. 288; Uéno 1966: fig. 5). A single male of this species was examined (MHNW) to establish the distributional record reported below.

#### Distribution and habitat.

**Figure 6.**
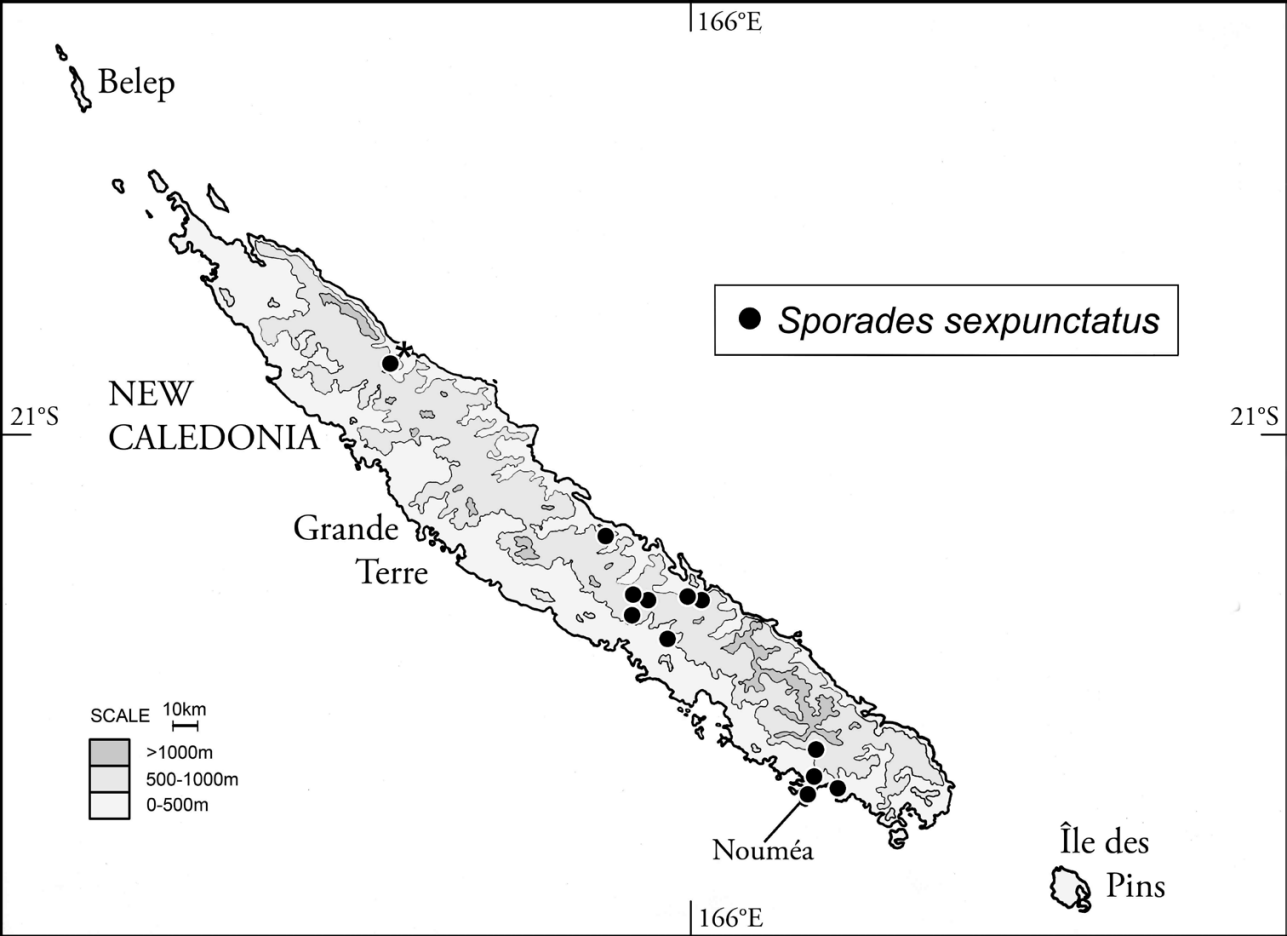
Distributional records for *Sporades sexpunctatus*; asterisked locality first reported herein.

Giachino (2012) summarized the distributional records of *S. sexpunctatus* based on examined material, showing that this species maintains a broad geographical distribution across the southern half of Grande Terre (Giachino 2012: fig. 11). Summarizing all published distributional records for this species, easily diagnosed by the male aedeagus (Fauvel 1882, 1903, Jeannel 1926, Uéno 1966, Donabauer 2011, Giachino 2012), results in a distributional estimate based on 29 specimens (Fig. 6). To these can be added one male: New Caledonia, N-Prov., Tendo 10 km WSW Hienghène, 20°42'46"S 164°48'56"E, 2.IX.1970, leg. H. Franz (M. Jäch pers. comm.). This most-recently documented locality extends the known distribution of *S. sexpunctatus* well northward in the Northern Province of Grande Terre. The known localities range in elevation 20–390 m, with the species repeatedly collected at sites above 300 m elevation: e.g., Col d'Amieu, Table Unio on the Serraméa-Canala Road, Parc des Grandes Fougères, near Thio, and on Mt. Koghi (Giachino 2012). Beetles have been collected at the margins of small streams, along a small stream in a ravine, and by splashing gravel bars along streams at night or during the day.

## Discussion

*Sporades jaechi* represents the fifth species of sg. Perileptosporades. Of the five species, *S. theryi* deviates most strongly based on larger body size, a different aedeagal median lobe configuration, a shorter, broader apex, and an extensive setal pelage on head, pronotum, and elytra (Deuve 2010). These deviations support *S. theryi* as the sister-group to the other four species. *Sporades theryi* is also one of the *Perileptosporades* involved in a sympatric species pair; it is sympatric with *S. modestior* at Pocquereux (Fig. 5). The other four species allopatrically divide Grande Terre, suggesting their speciation has been localized enough and recent enough to preclude development of secondary sympatry, or that sampling has been insufficient to discover sympatric populations. They are similar enough morphologically to suggest caution toward proposing a hypothesis of relationship. The various combinations of aedeagal median lobe configuration, the presence or absence and development of a dorsal setal pelage on head, pronotum or elytra, plus differential development of the recurrent stria may be informative, though no congruent hierarchy of character states strongly suggests a single phylogenetic hypothesis.

Jeannel (1926) compared *Trechodes* Blackburn and *Sporades*, and found them most similar among trechodine genera that he treated based on:

1. presence of the elytral basal groove;
2. the narrow, spindle-shaped apical maxillary palpomere contrasted to the broader, medially expanded penultimate segment;
3. a flat pronotum with mediobasal projection;
4. a deep sutural stria that is smoothly joined to the basal groove laterad the scutellum; and
5. a broadly widened aedeagal median lobe apex.

He noted that the two genera could be distinguished by the relatively short parameres of *Trechodes* spp. versus the robust, elongate parameres of *Sporades sexpunctatus*, the only species known at the time.

Also, the pronotum of *Trechodes* spp. is markedly protuberant medially along the pronotal base, with the pronotal hind angles situated well forward of the median base. *Trechodes* attains a very broad geographic distribution, ranging from Australia, the Philippines, Southeast Asia, Madagascar, and Africa (Jeannel 1926), with distributions of subsequently described species falling within that realm: *T. sicardi*, Madagascar (Alluaud 1932); *T. lepesmei*, Cameroon (Villiers 1940); *T. vadoni*, Madagascar (Jeannel 1946); *T. leleupi*, Congo (Basilewsky 1950); *T. katanganus*, Congo (Basilewsky 1958); *T. jeanneli*, Madagascar (Mateu 1958); *T. crypticus* and *T. lustrans*, Australia (Moore 1972); *T. daffneri*, Zaire (Casale 1986); *T. leclerci*, Thailand (Deuve 1987); *T. palawanensis*, the Philippines (Deuve 2001); *T. satoi*, Thailand (Uéno 1990); *T. laophilus*, Laos (Deuve 2002); and *T. lucanerii*, Ethiopia (Magrini et al. 2005). Maddison et al. (2019) reported an undescribed species from India. The morphological interpretation of *Trechodes* has remained remarkably constant across this series of authors describing species from tropical areas spanning 140° latitude. In all species, the male protarsomeres have the basal two segments denticulately expanded lateroapically (e.g., Jeannel 1926, Deuve 2001), a plesiomorphic condition shared with all Trechodina except *Sporades*, which have only the basal protarsomere expanded and bearing squamose setae ventrally.

The aedeagus of *Trechodes* spp. is characterized by an elongated median lobe with a narrowly attenuated apex, the tip of that apex bearing a hook, knob, or another type of curved appliance. For many of the species, the presence of spines on the internal sac is documented (e.g., Moore 1972, Casale 1986, Deuve 1987, 2001, Uéno 1988, 1990). All species exhibit a profound mediobasal pronotal protuberance, and polished glossy cuticle often with light and dark contrasting transverse elytral bands (e.g., Casale 1986; fig. 2; Deuve 1987: fig. 3; Uéno 1990 fig. 1).

Monophyly of *Sporades* relative to *Trechodes* is established based on the derived condition of only the basal male protarsomere dilated and bearing ventral squamose setae, and the presence of a fine setal pelage on the elytra (Uéno 1966), that pelage also variously present on the head or pronotum among the species (Deuve 2010, Donabauer 2011, Giachino 2012). Conversely, the uniform configuration of the pronotum in *Trechodes* spp. with its medially protuberant base has been used to support its monophyly relative to *Sporades*. Among the 12 *Sporades* spp., the derived, broadly rounded aedeagal median lobe apex observed in males of *M. jaechi* (Fig. 4) is shared with other members of sg. Perileptosporades: *S. millei*, *S. modestior*, *S. schuhi*, and *S. theryi*. This character in conjunction with the medial tooth in the labral excavation, and placement of the lateral seta far forward on the pronotum establishes *Perileptosporades*' monophyly. The remaining seven species consigned to the nominate subgenus Sporades exhibit a more heterogeneous range of genitalic characters. The generotypic *S. sexpunctatus* exhibits a uniquely inflated and twisted male median lobe apex (Jeannel 1926), whereas *S. macrops* (Uéno 1966) and *S. daccordii* (Giachino 2012) exhibit an elongate median lobe with an apical knob just apicad the ostial opening. In contrast, *S. testaceus* exhibits a very narrow, short, and apically rounded median lobe that barely extends beyond the parameral setae (Uéno 1966). *Sporades perileptoides* (Donabauer 2011) and *S. beatricis* (Giachino 2012) have a broader, foreshortened median lobe with a very narrow, rounded apex. And finally, the median lobe of *S. tachysoides* (Deuve 2010) is extremely foreshortened and broadly rounded apically. That so much variation occurs within the New Caledonian radiation of these species, whereas the aedeagus of *Trechodes* spp. consistently exhibits an apically attenuated and apically modified configuration across much of the Palaeotropical region begs explanation.

Recent molecular systematic results (Maddison et al. 2019) that include six *Trechodes* spp. plus *S. sexpunctatus* among 259 species representing 99 described genera of Trechitae find the two genera most closely related. Indeed, given the published taxonomic sampling scheme the single *Sporades* representative is the sister taxon to the Australian species pair, *T. bipartitus* (MacLeay) and *T. secalioides* (Blackburn); although bootstrap support for these relationships is not strong and more complete taxonomic sampling is called for. These three species, in turn, are the sister group to the species pair, *T. marshalli* Jeannel of Africa and *T. jeanneli* Mateu of Madagascar. The last two *Trechodes* spp. in the analysis, *T. alluaudi* Jeannel of Madagascar and *T.* sp. “India: Karnataka”, are successive sister groups to the previously mentioned species. The phylogenetic relationships of these taxa embrace the Indian Ocean, consistent with Jeannel's hypothesis that *Trechodes* was vicariated via the fragmentation of Gondwana: “Les *Trechodes* sont les restes de la vieille faune tropicale des débris du continent de Gondwana ... (Jeannel 1926: 487)”. However, the molecular systematic hypothesis whereby *Sporades* is derived from within the eastern, Australian *Trechodes* fauna, consistent with the suggestion that *Sporades* “may be regarded as a subgenus (Uéno 1966, p. 29)” of *Trechodes*, suggests that the putatively derived morphological characters supporting monophyly of *Trechodes* relative to *Sporades*, e.g., protruding mediobasal lobe of the pronotum, elongate male aedeagal median lobe with attenuated apex, relatively short male parameres, are either incorrectly polarized, or may transform to states present within *Sporades*. Certainly the male aedeagal median lobe characters might be so transformed, as the elongate lobe with an apical knob observed in *S. macrops* and *S. daccordii* is exceedingly similar to the aedeagus of *Trechodes marshalli* (Basilewsky 1958, fig. 13a). Thus, with regard to aedeagal characters, *T. marshalli* may serve as the phylogenetic nexus between *Trechodes* and *Sporades*. Short parameres relative to overall median lobe length have also been proposed to diagnose *Trechodes* (Jeannel 1926: 486), however the parameres of *T. daffneri* (Casale 1986: fig. 3B) are more elongate and not dissimilar in relative length to those of *Sporades* spp. The extensive aedeagal evolution within *Sporades* could thus be derived via modification of these various *Trechodes* configurations. Also, the mediobasal pronotal protuberance of *Trechodes* is an extreme version of the moderately lobate condition observed in *S. tachysoides* and *S. perileptoides*. Species of sg. Perileptosporades exhibit an even shorter mediobasal marginal expansion (e.g., Fig. 1); this too could represent a further derivation relative to the pronotal configurations of *Trechodes* and *Sporadess. s.* By this interpretation, the lobate *Trechodes* pronotum is plesiomorphic in this area of the cladogram, and reduction of the basal lobe is a derivation restricted to *Sporades*.

This contribution maintains the taxonomic independence of *Trechodes* and *Sporades* in the face of ambiguously conflicting morphological and molecular phylogenetic evidence. Both genera are presently diagnosable via observable morphological characters, although the polarity of their attendant character states may require reassessment in light of additional phylogenetic data from all sources. Should these two genera be synonymized in order to recognize monophyletic taxa, the geographically widespread *Trechodes* Blackburn, 1901 must fall into synonymy under the New Caledonian precinctive *Sporades* Fauvel, 1882. Such a happenstance illustrates that taxonomic nomenclature operates by stable rules (I.C.Z.N. 1999), whereas phylogenetic analysis must respond to the preponderance of data. This contribution continues the dogged pursuit of trechodine diversity that stretches back to Fauvel (1882), Blackburn (1901), and Jeannel (1926); that pursuit a recurring first step toward hypothesizing patterns of evolution for the group.

## Supplementary Material

XML Treatment for Sporades (Perileptosporades) jaechi

XML Treatment for Sporades (s. s.) sexpunctatus
